# Prevalence of Lung Metastases among 19,321 Metastatic Colorectal Cancer Patients in Eight Countries of Europe and Asia

**DOI:** 10.3390/curroncol28060423

**Published:** 2021-11-30

**Authors:** Markus S. Jördens, Simon Labuhn, Tom Luedde, Laura Hoyer, Karel Kostev, Sven H. Loosen, Christoph Roderburg

**Affiliations:** 1 Department of Gastroenterology, Hepatology and Infectious Diseases, University Hospital Düsseldorf, Medical Faculty of Heinrich Heine University Düsseldorf, 40225 Düsseldorf, Germany; simon.labuhn@med.uni-duesseldorf.de (S.L.); luedde@hhu.de (T.L.); Sven.Loosen@med.uni-duesseldorf.de (S.H.L.); Christoph.Roderburg@med.uni-duesseldorf.de (C.R.); 2 Epidemiology, IQVIA, 60549 Frankfurt, Germany; laura.hoyer@iqvia.com (L.H.); Karel.Kostev@iqvia.com (K.K.)

**Keywords:** CRC, colorectal, lung metastases, prevalence

## Abstract

Background: Colorectal cancer is one of the most common malignancies in the Western world, and is responsible for about 10% of annual cancer-related deaths. Especially for UICC stage IV, the probability of survival is significantly reduced. Little is known about risk factors for specific metastatic patterns of colorectal cancer that may also influence patients’ overall survival. Methods: We used data from the IQVIA oncology dynamics (OD) database to determine the prevalence of pulmonary metastases in 19,321 patients with UICC stage IV colorectal cancer in eight European and Asian countries. Results: In total, 6132 of 19,321 (31.7%) study patients had lung metastases, with a higher prevalence among patients with rectal (37.5%) than colon (30.1%) cancer. When compared to China as the country with the lowest lung metastases prevalence, the odds for lung metastases were highest in UK (OR: 2.02, 95%CI: 1.80–2.28), followed by Italy (OR: 1.86, 95%CI: 1.52–2.27), Spain (OR: 1.85, 95%CI: 1.64–2.09), and Germany (OR: 1.47, 95%CI: 1.26–1.71). Conclusion: The prevalence of pulmonary metastases in UICC stage IV colorectal cancer varies widely among the different analyzed countries. Although the present data are purely descriptive, a possible combination of ethnic, environmental, and health care system-associated differences could be discussed as the underlying cause. Further studies are needed to investigate the reasons for differences in the prevalence of lung metastases.

## 1. Introduction

In recent decades, colorectal cancer has become one of the most common tumors in the Western world, with a lifetime disease risk of 3–5%, and most recently, accounted for approximately 10% of annual cancer deaths [1]. Unfortunately, approximately one quarter of patients are diagnosed at a metastatic stage, which complicates curative therapeutic approaches [2]. The most common site of metastasis is the liver, followed by the lung [3]. Several studies estimate that 6–8% of colon carcinomas and 10–18% of rectal carcinomas metastasize to the lung [4,5]. Interestingly, the pattern of metastasis appears to influence overall survival in colon carcinoma. For example, one study demonstrated improved survival with lung-only metastases compared to liver-only metastases [6]. While risk factors for the development of colon cancer are well studied, there are little data to date on specific risk factors for particular metastatic patterns [1]. In our retrospective work, we looked at differences in the prevalence of pulmonary metastases in Union internationale contre le cancer (UICC) stage IV colon cancer in different countries to identify risk factors for pulmonary tumor spread.

## 2. Methods

### 2.1. Database

This retrospective cross-sectional study is based on the data from the IQVIA oncology dynamics (OD) database [7,8,9]. This source is a cross-sectional semi-retrospective survey collecting anonymized patient cases from a representative panel of oncologists. OD collects fully anonymized patient-level data on drug-treated cancer cases in several countries worldwide. Data collection and reporting are conducted through a standardized online questionnaire where all items are mandatory. A reporting manual with precise instructions on filling out the questionnaire is provided to each respondent. Specific instructions are displayed through a ‘pop-up’ system throughout the survey to provide clear definitions for the desired variables. Physicians are also asked to enter factual information from the patient medical record to avoid recall biases. Further tactics to ensure input accuracy include controlled code lists and multiple-choice questions, as well as interactive filters that limit non-applicable questions (e.g., items on cancer-specific biomarkers). Responses are immediately validated against previous answers and reference files; “unexpected value” messages are displayed to the participant, prompting them to double-check their response. Physicians are instructed to report the most recent consecutive cases (up to 20 cases depending on the specialty) that they had treated during the last 7-day period to discourage selective case submission. After the form submission, additional validations and trend checks are performed; anomalous values are discussed with the participant who had submitted them, and are corrected as needed.

### 2.2. Patient Selection and Study Outcome

Surveys of patients with either colon (ICD-10: C18) or rectum (ICD-10: C20) cancer in the stage IV (distant metastases) filled in the time between 1 January 2017 and 31 March 2021. Countries which data were available for were Germany, France, United Kingdom (UK), Spain, Italy, China, Korea, and Japan. The outcome of the study was the proportion of cancer patients with a documentation of lung metastasis depending on country, age, sex, and co-diagnosis of chronic obstructive lung disease (COPD, ICD-10: J44).

### 2.3. Statistical Analysis

The prevalence of lung metastases was calculated as the proportion of patients with lung metastases on all patients with stage IV, and was shown by country. To investigate the lung metastasis probability, a multivariable logistic regression model was fitted with lung metastases (yes/no) as dependent variable and age, sex, cancer type, COPD co-diagnosis, and country as impact variable. The results of the regression analyses are presented as odds ratios (ORs) with 95% confidence intervals (CIs). *p*-values lower than 0.05 were considered statistically significant. All analyses were performed using SAS 9.4 (SAS Institute, Cary, NC, USA).

## 3. Results

### 3.1. Baseline Characteristics of Study Population

Overall, 19,321 people with metastatic colorectal cancer (15,086 colon and 4235 rectal cancer) documented by 2122 physicians were included in this study. The baseline characteristics of the study sample and the patient population analyzed in each country are displayed in Table 1. Mean age was 65.2 years, and 61.4% were males. Moreover, 18% of study patients were treated in Italy, followed by China (15.7%), Germany (15.5%), and France (13.4%).

### 3.2. Prevalence of Lung Metastases among Patients with Metastatic Colorectal Cancer

**Figure 1:**
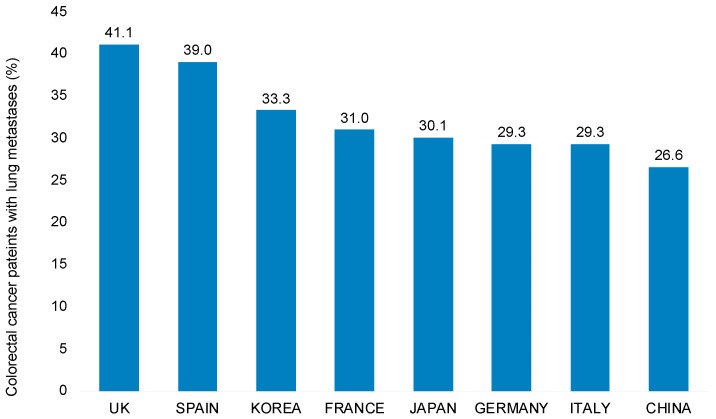
Prevalence of lung metastases among patients with UICC stage IV metastatic colorectal cancers in eight countries of Europe and Asia.

In total, 6132 of 19,321 (31.7%) study patients had lung metastases. However, only 784 patients had only lung and no other distant metastases, as most patients had several metastases, including lung metastases. The prevalence of lung metastases was higher among patients with rectal (37.5%) than colon (30.1%) cancer. Figure 1 shows the prevalence of lung metastases in colorectal cancer patients in eight countries. This prevalence was highest in the United Kingdom (41.1%), followed by Spain (39.0%) and Korea (33.3%). The lowest prevalence was observed in China (26.6%).

### 3.3. Results of Multivariable Regression Analysis

In a multivariate regression model, rectal cancer (OR: 1.52, 95%CI: 1.42–1.64, *p* < 0.001) was significantly associated with higher odds of lung metastases, compared to colon cancer as a reference group (Table 2). Furthermore, COPD diagnosis was also significantly associated with lung metastases (OR: 1.22: 95%CI: 1.09–1.36). When compared to China as the country with the lowest lung metastases prevalence, the odds for lung metastases were highest in UK (OR: 2.02, 95%CI: 1.80–2.28), followed by Italy (OR: 1.86, 95%CI: 1.52–2.27), Spain (OR: 1.85, 95%CI: 1.64–2.09), and Germany (OR: 1.47, 95%CI: 1.26–1.71).

## 4. Discussion

Lung metastases are the second most common metastatic site in colorectal carcinoma, with studies recently demonstrating marked differences in the metastatic pattern between colon and rectal carcinoma [10,11]. Furthermore, there appears to be an additional dependence on histologic subtype as to which organs are preferentially invaded by metastases [12].

In our study, we investigated the prevalence of lung metastases in UICC stage IV colorectal cancer in different countries. First, we found an increased incidence of pulmonary metastases in rectal cancer, as expected from the different metastatic patterns. This is in line with several other studies, identifying higher odds of lung metastases in rectal cancer [12]. Furthermore, we found an increased risk of lung metastases in patients with existing COPD. This may be related to existing data, showing that smokers have an increased risk of pulmonary metastases in colorectal carcinoma [13]. In addition, worsened survival of patients with COPD and colorectal cancer has been demonstrated, potentially as a result of inflammatory cytokine driven tumor cell growth [14,15,16,17].

More difficult to assess are the marked prevalence differences between the different countries studied. On the one hand, ethnic differences cannot be solely responsible for the different prevalences, since both low prevalences of lung metastases (Italy and China) and high prevalences (UK, Korea) occur in the Caucasian population, as well as in the Asian population. Nevertheless, the difference between the leading countries UK and Spain and the rest of the studied countries is pronounced, thus contributing ethnical or genetical factors cannot be excluded, as the odds ratio of lung metastases for all Asian countries is fairly low compared to the European ones. The assessment regarding environmental risk factors is similarly difficult. Smoking appears to increase the risk of lung metastases in CRC, yet the prevalences of tobacco smoking between China (27.7% in 2015) and the UK (14.1% in 2019) or Germany (29.7% in 2011) cannot explain the prevalence differences in lung metastasis at all [18,19]. Furthermore, air pollution cannot explain these differences, as pollution in China has increased in recent years and is higher than in other developed countries, arguing for highest prevalence rates in China [20].

Another issue might be differences in health care, and as a consequence, the detection of lung metastasis in different countries. Thus, it could be argued that, due to better health care systems and surveillance, longer survival of patients and therefore the more frequent occurrence of lung metastases would be reasonable, but the quality of health care should be accounted similar at least in the European countries studied and thus alone cannot explain the different prevalences.

Overall, our data are purely descriptive, reflecting the prevalence of pulmonary metastases in CRC in different countries. The cause of the differences in distribution cannot be ascertained from our data, but can only be discussed in the context of various influencing factors such as environmental risk factors, ethnic differences and differences in health care systems. Furthermore, treatment- and health care- associated factors, like elective or urgent operation, type of chemotherapeutic regimen or hospital volume, are not included in our database, and can influence our results. Our data should be the basis for prospective studies in this field to identify further risk factors for lung metastases in CRC, in order to develop better prognostic models, but also to improve risk assessment and reduction in the long term.

## 5. Conclusions

The prevalence of pulmonary metastases in UICC stage IV colorectal cancer varies among different countries. We suggest a possible combination of ethnic, environmental, and health care system-associated differences as the underlying cause.

## Figures and Tables

**Table 1:** Baseline characteristics of study patients.

Variable	N (%)
N	19,321
Cancer type	
Colon	15,086 (78.1)
Rectum	4235 (21.9)
Age (mean, SD)	65.2 (11.3)
Males (%)	11,863 (61.4)
Facility	
Hospital	13,903 (72.0)
Office-based oncologists	1930 (10.0)
Unknown	3488 (18.0)
Co-Diagnosis of COPD	1732 (9.0)
Country	
Germany	2988 (15.5)
France	2594 (13.4)
Italy	3486 (18.0)
Spain	2203 (11.4)
UK	2224 (11.5)
Japan	1713 (8.9)
Korea	1074 (5.6)
China	3039 (15.7)

**Table 2:** Association between age, sex, country, and the prevalence of lung metastases in UICC stage IV metastatic colorectal cancer patients (multivariable logistic regression model).

Variable	OR (95%CI)	*p* Value
Age		
Age ≤ 50	Reference	
Age 51–60	1.04 (0.92–1.17)	0.571
Age 61–70	1.09 (0.97–1.21)	0.157
Age > 70	1.09 (0.97–1.22)	0.154
Sex		
Male sex	0.98 (0.92–1.04)	0.444
Female sex	Reference	
Cancer type		
Colon cancer	Reference	
Rectum cancer	1.52 (1.42–1.64)	<0.001
COPD	1.22 (1.09–1.36)	<0.001
Country		
Germany	1.47 (1.26–1.71)	<0.001
France	1.29 (1.15–1.46)	<0.001
Italy	1.86 (1.52–2.27)	<0.001
Spain	1.85 (1.64–2.09)	<0.001
UK	2.02 (1.80–2.28)	<0.001
Japan	1.20 (1.05–1.37)	0.008
Korea	1.28 (1.10–1.50)	0.002
China	Reference	

## Data Availability

Data are available upon request from the Department of Gastroenterology, Hepatology and Infectious Diseases of the University Hospital Düsseldorf for researchers who meet the criteria for access to confidential data: Wissenschaft.Gastro@med.uni-duesseldorf.de.

## References

[1] Kuipers E.J., Grady W.M., Lieberman D., Seufferlein T., Sung J.J., Boelens P.G., Van De Velde C.J.H., Watanabe T. (2015). Colorectal cancer. Nat. Rev. Dis. Primers.

[2] Brouwer N.P., Bos A.C., Lemmens V.E., Tanis P., Hugen N., Nagtegaal I., De Wilt J.H., Verhoeven R. (2018). An overview of 25 years of incidence, treatment and outcome of colorectal cancer patients. Int. J. Cancer.

[3] Edwards B.K., Ward E., Kohler B.A., Eheman C., Zauber A.G., Anderson R.N., Jemal A., Schymura M.J., Lansdorp-Vogelaar I., Seeff L.C. (2010). Annual report to the nation on the status of cancer, 1975–2006, featuring colorectal cancer trends and impact of interventions (risk factors, screening, and treatment) to reduce future rates. Cancer.

[4] Tan K.K., Lopes Gde L., Sim R. (2009). How uncommon are isolated lung metastases in colorectal cancer? A review from database of 754 patients over 4 years. J. Gastrointest. Surg..

[5] Parnaby C.N., Bailey W., Balasingam A., Beckert L., Eglinton T., Fife J., Frizelle F.A., Jeffery M., Watson A.J.M. (2012). Pulmonary staging in colorectal cancer: A review. Color. Dis..

[6] Wang J., Li S., Liu Y., Zhang C., Li H., Lai B. (2019). Metastatic patterns and survival outcomes in patients with stage IV colon cancer: A population-based analysis. Cancer Med..

[7] Zhao Z., Pelletier E., Barber B., Bhosle M., Wang S., Klingman D., Gao S. (2012). Major Surgery in Patients with Metastatic Colorectal Cancer in Western Europe. J. Gastrointest. Cancer.

[8] Marchetti P., Maass N., Gligorov J., Berger K., MacDougall F., Montonen J., Lewis J. (2017). Patient database analysis of fulvestrant 500 mg in the treatment of metastatic breast cancer: A European perspective. Breast.

[9] Chambers P., Man K., Lui V.W., Mpima S., Nasuti P., Forster M.D., Wong I.C. (2020). Understanding Molecular Testing Uptake Across Tumor Types in Eight Countries: Results From a Multinational Cross-Sectional Survey. JCO Oncol. Pract..

[10] Weiss L., Grundmann E., Torhorst J., Hartveit F., Moberg I., Eder M., Fenoglio-Preiser C.M., Napier J., Horne C.H.W., Lopez M.J. (1986). Haematogenous metastastic patterns in colonic carcinoma: An analysis of 1541 necropsies. J. Pathol..

[11] Qiu M., Hu J., Yang D., Cosgrove D.P., Xu R. (2015). Pattern of distant metastases in colorectal cancer: A SEER based study. Oncotarget.

[12] Riihimäki M., Hemminki A., Sundquist J., Hemminki K. (2016). Patterns of metastasis in colon and rectal cancer. Sci. Rep..

[13] Yahagi M., Tsuruta M., Hasegawa H., Okabayashi K., Toyoda N., Iwama N., Morita S., Kitagawa Y. (2017). Smoking is a risk factor for pulmonary metastasis in colorectal cancer. Color. Dis..

[14] Van de Schans S., Janssen-Heijnen M., Biesma B., Smeenk F., van de Poll-Franse L., Seynaeve C., Coebergh J. (2007). COPD in cancer patients: Higher prevalence in the elderly, a different treatment strategy in case of primary tumours above the diaphragm, and a worse overall survival in the elderly patient. Eur. J. Cancer.

[15] Chen Y.-C., Li M.-C., Yu Y.-H., Lin C.-M., Wu S.-Y. (2021). Chronic Obstructive Pulmonary Disease and Its Acute Exacerbation before Colon Adenocarcinoma Treatment Are Associated with Higher Mortality: A Propensity Score-Matched, Nationwide, Population-Based Cohort Study. Cancers.

[16] Barnes P.J., Celli B.R. (2009). Systemic manifestations and comorbidities of COPD. Eur. Respir. J..

[17] Coussens L.M., Werb Z. (2002). Inflammation and cancer. Nature.

[18] Lampert T., von der Lippe E., Muters S. (2013). Prevalence of smoking in the adult population of Germany: Results of the German Health Interview and Examination Survey for Adults (DEGS1). Bundesgesundheitsblatt Gesundh. Gesundh..

[19] Parascandola M., Xiao L. (2019). Tobacco and the lung cancer epidemic in China. Transl. Lung Cancer Res..

[20] Guan W.-J., Zheng X.-Y., Chung K.F., Zhong N.-S. (2016). Impact of air pollution on the burden of chronic respiratory diseases in China: Time for urgent action. Lancet.

